# Efficiency of Health Care Production in Low-Resource Settings: A Monte-Carlo Simulation to Compare the Performance of Data Envelopment Analysis, Stochastic Distance Functions, and an Ensemble Model

**DOI:** 10.1371/journal.pone.0147261

**Published:** 2016-01-26

**Authors:** Laura Di Giorgio, Abraham D. Flaxman, Mark W. Moses, Nancy Fullman, Michael Hanlon, Ruben O. Conner, Alexandra Wollum, Christopher J. L. Murray

**Affiliations:** Institute for Health Metrics and Evaluation, University of Washington, Seattle, WA, United States of America; Universidad de Las Palmas de Gran Canaria, SPAIN

## Abstract

Low-resource countries can greatly benefit from even small increases in efficiency of health service provision, supporting a strong case to measure and pursue efficiency improvement in low- and middle-income countries (LMICs). However, the knowledge base concerning efficiency measurement remains scarce for these contexts. This study shows that current estimation approaches may not be well suited to measure technical efficiency in LMICs and offers an alternative approach for efficiency measurement in these settings. We developed a simulation environment which reproduces the characteristics of health service production in LMICs, and evaluated the performance of Data Envelopment Analysis (DEA) and Stochastic Distance Function (SDF) for assessing efficiency. We found that an ensemble approach (ENS) combining efficiency estimates from a restricted version of DEA (rDEA) and restricted SDF (rSDF) is the preferable method across a range of scenarios. This is the first study to analyze efficiency measurement in a simulation setting for LMICs. Our findings aim to heighten the validity and reliability of efficiency analyses in LMICs, and thus inform policy dialogues about improving the efficiency of health service production in these settings.

## Introduction

The *World Health Report 2010* estimates that 20% to 40% of all health spending is currently wasted through inefficiency [1]. This is particularly striking considering how few financial resources are available in many countries [2]. However, no consensus exists on the most appropriate models and methods for estimating efficiency across settings, and there are robust and ongoing debates around two major methodological approaches: Data Envelopment Analysis (DEA) and Stochastic Frontier Analysis (SFA) [3]. The result of such debate has important implications, as DEA and SFA can yield very different estimates of health facility efficiency [3]. Therefore, validating an accurate method to measure the efficiency of health facilities in low- and middle-income countries (LMICs) is a pressing need.

One way to compare the accuracy of competing methods for measuring efficiency is through simulation studies. In a simulation study, we create the dataset on which we run the efficiency measurement method and use a data generation process designed specifically to test the method. In this simulation setting, we know the true efficiency of each health facility, and, for any method for estimating efficiency, we can compare the estimated value to the truth, and precisely quantify the level of bias and error in the corresponding predictions. This allows us to compare methods and to quantify in absolute terms how accurately each method performs [4].

Simulation studies have been used extensively in previous research to validate and compare methods for efficiency measurement (in a wide variety of industries, not just health facility production) [5–12], but they typically assume production characteristics of competitive markets, which may not generalize to health service production in LMICs. For example, previous studies assume that the majority of firms operate relatively efficiently (a right-skewed distribution for efficiency) [5,7‒10]; the production process is appropriately represented by a Cobb-Douglas or piecewise Cobb-Douglas production function [6,7,10]; and that firms use all possible inputs and produce all possible outputs in service production [5,6,13]. In addition, most simulation studies analyze production functions involving a single output [5,8,10], as they are relatively easier to estimate; notable exceptions have been simulation studies measuring efficiency of education systems [14]. More recent efforts involve simulation studies with more flexible production functions and multiple-outputs processes [5,8,15], but they generally focus on simulating efficiency in competitive markets.

In this study, we developed a simulation environment that captures the important aspects of health facilities in a LMIC setting. We included three key differences from prior simulations: (1) multiple-output production functions, other than Cobb-Douglas; (2) efficiency drawn from a highly dispersed distribution; and (3) a subset of facilities with only a subset of the possible inputs available (nurses, doctors, beds) or only a subset of the possible outputs (outpatient visits, births, anti-retroviral therapy [ART] visits). We then applied DEA and the multiple-output implementation of SFA, Stochastic Distance Function (SDF), to our simulated datasets and assessed their respective performance. Since neither approach performed to our satisfaction, we then developed and tested a novel extension to DEA that incorporated data-driven restrictions on the allowed transformation weights, and a novel ensemble model of restricted versions of DEA and SDF.

## Materials and Methods

### Efficiency measurement approaches

#### DEA, weights, and restrictions

Evaluating facility efficiency of service production requires comparing facilities across multiple dimensions, including several inputs and outputs. DEA defines a composite performance indicator by computing the ratio of weighted outputs to weighted inputs [16]. Facilities with the highest ratios of outputs to inputs are considered the best performing, and are assigned an efficiency score equal to one. All other facilities receive an efficiency score reflecting their relative performance to the frontier set by these best-performing facilities [17,18].

Assuming that there are a total of *n* facilities (also known as decision-making units, or DMUs), facility *i* (where *i* ranges from 1 to *n*) uses an amount *x*_*ri*_ of input *r* (where *r* ranges from 1 to *R*) and produces an amount *y*_*ji*_ of output *j* (where *j* ranges from 1 to *J*). For each facility *i*, DEA identifies input and output weights, *v*_*ri*_ and *u*_*ji*_, which maximize the efficiency score, $\theta_{i}^{\text{DEA}}$, as defined in Eq (1):

$$\begin{array}{l} \text{max}\;\theta_{i}^{\text{DEA}}=\sum_{j=1}^{J}u_{ji}y_{ji}/\sum_{r=1}^{R}v_{ri}x_{ri}\\ \;s.t.\;\sum_{j=1}^{J}u_{ji}y_{ji}\;/\sum_{r}^{R}v_{ri}x_{ri}\;\leq 1\;\text{for}\;i=1,\ldots \;,\;n\\ \;v_{ri},\;u_{ji}\geq 0\;\text{for all}\;r\;\text{and}\;j. \end{array}$$ (1)

This mathematical problem is solved under two constraints. The first constraint states that the efficiency score of any DMU in the sample must be less than or equal to one when the optimal weights of a given DMU are applied to its inputs and outputs. The second constraint restricts the weights to be non-negative.

Our formulation of DEA uses constant returns to scale (CRS) and an output orientation. CRS stipulates that changes in output production are proportional to changes in all inputs. A slightly more complicated version of DEA uses a variable returns to scale (VRS) assumption to reflect the fact that the production technology may exhibit increasing, decreasing, or CRS. This assumption is modeled by adding an additional parameter to the mathematical problem shown in Eq (1) [16]. Another variant of DEA takes an input orientation, which changes the interpretation of the efficiency score. An output-oriented model seeks to increase outputs given its current inputs and an input-oriented model aims to minimize the use of inputs given its current outputs. We prefer the output orientation, as expanding outputs (e.g., the number of health services provided) is a goal in LMICs, and health facility managers often have limited control over inputs (e.g., the number of doctors at the facility). The same perspective has been applied in previous studies of health facility efficiency in LMICs [19,20].

DEA’s principal advantage is its non-parametric nature, as it aims to find the unique set of input and output weights for each *i*^*th*^ DMU that maximizes $\theta_{i}^{\text{DEA}}$ [16]. In doing so DEA may assign weights of zero to critical inputs or outputs implying undefined rates of substitution or transformation [21]. Accordingly, many innovative methods preserve interpretability by imposing weight restrictions [16], such as absolute weight restrictions, cone ratio model, relative weight restrictions, and restrictions on virtual inputs and outputs. In the present study we developed a novel extension of relative weight restrictions.

Relative weight restrictions consist of placing lower- (*L*) and upper- (*U*) bounds on the ratio of weights of each output *j* to output 1 and each input *r* to input 1 as seen in Eq (2):

$$\begin{array}{c} L_{j}\leq u_{ij}/u_{i1}\leq U_{j}\\ L_{r}\leq v_{ir}/v_{i1}\leq U_{r} \end{array}$$ (2)

Previous work has defined *L* and *U* bounds with subjective expert opinions [17,21], or by input wages and output prices that may not be available for health facilities in LMICs. With our novel relative weight restriction approach, hereafter referred to as restricted DEA (rDEA), we first performed unrestricted DEA, and then used the first step DEA weights to inform second step rDEA restrictions. Specifically, we used non-zero weights calculated in DEA to form a distribution of relative weights for each of the *R* − 1 inputs and *J* − 1 outputs, relative to the first input and output. From these distributions, we then drew lower and upper *p*-percentiles from the relative weight distributions to set *L* and *U* bounds.

The performance of rDEA depends on the percentile *p*, so we used our baseline simulation scenario with a range of *p*-percentiles to determine *p*.

For all DEA-based models (DEA and rDEA), to detect and remove outliers arising due to noise, we conducted a super-efficiency analysis, where the efficiency score of each DMU was calculated based on the frontier estimated from all other DMUs (which can yield efficiency scores that exceed 1) [16]. We iteratively ran this super-efficiency analysis until no DMUs’ super-efficiency score exceeded 1.5 or until 5% of DMUs were removed from the original sample. All DMUs detected in this super-efficiency analysis were assigned an efficiency score of one.

### Stochastic Distance Function

In advance, we caution the reader to note that the notation shared by DEA and SFA disciplines often is not in agreement. For the purposes of the present study we use notation consistent with each discipline. Unlike DEA, SDF requires an assumption regarding the functional form of the multiple-output production function and distribution of efficiency. The SDF approach is commonly used to estimate technical efficiency for production processes with multiple outputs [22,23]. While greater detail on SDF can be found elsewhere [24,25], we provide a brief overview of a Cobb-Douglas multiple-output production function. Eq (3) below shows a Cobb-Douglas multiple-output production function assuming full efficiency:

$$1=Ax_{1i}{}^{\beta_{1}}x_{2i}{}^{\beta_{2}}x_{3i}{}^{\beta_{3}}y_{1i}{}^{\alpha_{1}}y_{2i}{}^{\alpha_{2}}y_{3i}{}^{\alpha_{3}}$$ (3)

If we further assume that $\sum_{j=1}^{3}\;a_{j}=-1$, Eq (3) may be written with *y*_1_ on the left hand side [26]. We then may take the natural logarithm of Eq (3) and relax the assumption of full efficiency and allow for measurement error, which are both captured by the residual, *ε*_*i*_, to arrive at Eq (4) which may be estimated with SDF:

$$d\left(x,y\right)=-\text{ln}\;y_{1i}=\beta_{0}+\beta_{1}\;\text{ln}\;x_{1i}+\beta_{2}\;\text{ln}\;x_{2i}+\beta_{3}\;\text{ln}\;x_{3i}+\alpha_{2}\;\text{ln}\frac{y_{2i}}{y_{1i}}+\alpha_{3}\;\text{ln}\frac{y_{3i}}{y_{1i}}+\varepsilon_{i}$$ (4)

Hereafter we denote SDF estimation of a Cobb-Douglas multiple-output production function as SDF-CD. The residual in Eq (4) may be represented by *ε*_*i*_ = *v*_*i*_ − *u*_*i*_, with *v*_*i*_ denoting measurement error and *u*_*i*_ denoting inefficiency. The latter may be converted into a SDF technical efficiency score by $\theta_{i}^{\text{SDF}}=e^{-u}$. It follows that SDF techniques must disentangle inefficiency from measurement error [27] and it does so by assuming that the two components follow different distributions [28]. In most implementations of SDF, the random measurement error component is assumed to be normally distributed, $N\left(0,\sigma_{v}^{2}\right)$, while inefficiency is assumed to be right-skewed (usually half-normal). DMU-specific efficiency is usually computed by means of the JLMS estimator [27], which consists of calculating the expected mean value of inefficiency conditional upon the composite residual *ε*_*i*_, or *E*(*u*_*i*_|*v*_*i*_ + *u*_*i*_). The cumulative distribution of inefficiency is typically assumed to be half normal [27,29,30], for which the conditional mean is presented in Eq (5):

$$\text{E}(u_{i}|\varepsilon_{i})=\frac{\sigma \lambda }{1+\lambda^{2}}[\frac{\phi (\gamma_{i})}{1-\Phi (\gamma_{i})}-\gamma_{i}]$$ (5)

where $\sigma =\sqrt{\sigma_{u}^{2}+\sigma_{v}^{2}},\;\lambda =\frac{\sigma_{u}}{\sigma_{v}}\;,\;\gamma_{i}=\varepsilon_{i}\frac{\lambda }{\sigma }.$ *ϕ*(*γ*_*i*_) and Φ(*γ*_*i*_) denote the density and cumulative distribution of the standard normal. Note that we expect inefficiency to be very dispersed for health facilities in LMICs, and therefore this model may be misspecified; we will consider the effect of the misspecification in our simulation scenarios detailed below.

We used SDF-CD with half-normally distributed inefficiency, and since the logarithm is not defined for zero values, we used a small positive value (10^−10^) to replace any inputs or outputs with zero values [31]. Moreover, we used constrained optimization while estimating SDF-CD to impose economic interpretability conditions of $\frac{\partial \;\text{ln}\;d\left(x,y\right)}{\partial \;y_{j}}=\alpha_{j}>0$ and $\frac{\partial \;\text{ln}\;d\left(x,y\right)}{\partial \;x_{r}}=\beta_{j}<0$ [22,32], and to impose the restriction of *λ* > 0 to ensure interpretable variances of inefficiency or measurement error; hereafter we refer to the use of these restrictions for SDF-CD as restricted SDF-CD (rSDF-CD).

#### An ensemble modeling approach

Our ensemble model (ENS) consisted of combining efficiency estimates from rSDF-CD and rDEA. Efficiency estimates resulted from the mean score for each facility, as shown in Eq (6):

$$\theta_{i}^{\text{ENS}}=\frac{\theta_{i}^{\text{rSDF}-\text{CD}}+\theta_{i}^{\text{rDEA}}}{2}$$ (6)

This approach has been considered previously in a simulation scenario [33], which varies from our study as the analysis focused on single-output production functions and used traditional (unrestricted) DEA.

### Simulation design

#### Features of health service production in LMICs

Little is known as to whether the majority of facilities in LMICs are performing efficiently, particularly in health sectors that largely lack market mechanisms to support optimizing facility production behavior. By contrast, many higher-income countries feature some incentives to maximize service production, including payment systems linked to production and/or the presence of competition [34]. To capture large variations in facility input use and output production in LMICs, we applied a uniform distribution for efficiency. We also modeled multiple-output production functions, which permitted facilities to produce one, two, or three different outputs. This flexibility allowed for variations in treatment patterns across facilities and reflected the existence of zero inputs and outputs in facility-level datasets [35]. Lastly, we started with the simplest scenario of a linear multiple-output production function for health service provision and then varied this scenario to include traditional functional forms. The linear multiple-output production function is likely to approximate more complex, unknown non-linear multiple-output production functions, as well as serve as the simplest production scenario through which any increases in inputs result in rising outputs. The multiple-output production function in LMICs may be substantially different than those of high-income countries. First, higher and more variable rates of input substitutability may occur due to human resources shortages and redistribution of tasks among health workforce teams to overcome gaps in trained medical staff [36,37]. Second, for LMICs, increase sin inputs would likely result in more services provided, particularly since LMICs often face high rates of unmet demand amid resource shortages. By contrast, Cobb-Douglas production functions result in no output production if any input is set to zero.

### A novel simulation design for LMICs: baseline scenario

We modeled a multi-input, multi-output production function as shown in Eq (7). We assumed that production technology could be represented by the transformation of three discretionary inputs, *x*_1_, *x*_2_, and *x*_3_, into a total productive capacity *Y*, according to the following linear production function that satisfies CRS:

$$Y_{i}=0.2\;x_{1,\text{i}}+0.5\;x_{2,\text{i}}+0.3\;x_{3,\text{i}}$$ (7)

We also assumed that there was inefficient behavior, and that efficiency followed a uniform distribution, *θ*_*i*_ ∼ *unif*(0,1). The efficiency score scaled down the total productive capacity ($Y_{i}^{\prime }$) by, $Y_{i}^{\prime }=Y_{i}\cdot \theta_{i}$. Three inputs were drawn for a sample of 200 facilities from the following uniform distributions: *x*_1_ ∼ *unif*(0,5), *x*_2_ ∼ *unif*(0, 10), and *x*_3_ ∼ *unif*(0, 8). The total productive capacity was then used to produce up to three outputs. We assumed that all facilities produced output *y*_3_, while only a subset of facilities produced output *y*_1_ or output *y*_2_, or both (details on model output production are presented in S1 Appendix). This approach was used to reflect that, in reality, it is unlikely that every facility produces all possible outputs. For instance, based on a nationally-representative sample of health facilities in Zambia [38], 100% of health centers offered general outpatient services, but only 12% reported providing routine delivery services. For each output produced, we ascertained how much of the facility’s total output capacity was used for its production and assigned a productive capacity term $Y_{ji}^{\text{S}}$ (see details in S1 Appendix). Last, we assumed that the production volume of each observed output type ($y_{ji}^{\text{obs}}$) was dependent upon the resources needed to produce each output. This meant that given a facility’s set productive capacity, a lower output volume would be produced if a given output was more resource-intensive in its production (e.g., inpatient services in comparison with outpatient care). The final volume of outputs produced by a facility was defined in Eq (8), where we assumed that output *y*_3_ was the most resource-intensive to produce, followed by *y*_2_ and *y*_1_:

$$\begin{array}{r} y_{1,i}^{o\text{bs}}=Y_{1,i}^{\text{S}}/0.25\\ y_{2,i}^{\text{obs}}=Y_{2,i}^{\text{S}}/0.5\\ y_{3,i}^{\text{obs}}=Y_{3,i}^{\text{S}}\;/1.0 \end{array}$$ (8)

The main assumptions of this data generation process were varied in sensitivity analyses detailed below and summarized in Table 1. Efficiency scores were estimated for each simulation scenario using four approaches: DEA, rDEA, rSDF-CD, and ENS. For each simulation scenario we generated 2,000 independent replications. All models were estimated using the Benchmarking package available in the programming language R (version 3.1.2) [39]. Code used for this study is publicly available online and can be downloaded through the Global Health Data Exchange (GHDx): http://ihmeuw.org/eff_sim.

**Table 1:** Baseline simulation design for LMIC and variations.

Scenario	Description of baseline simulation design	Factors we varied
***a***	Define a sample size (number of DMUs)	Number of DMUs
***b*, *c***	Simulate inputs, *x*_1_ ∼ *unif*(0,5), *x*_2_ ∼ *unif*(0, 10), *x*_3_ ∼ *unif*(0, 8)	Correlation between inputs and fixed inputs
***d***	Simulate measurement error, $v_{i}∼N\left(0,\sigma_{v}^{2}\right)$	Type and variation of measurement error
***e***	Simulate efficiency, *θ*_*i*_ ∼ *unif*(0,1)	Distribution and variation of efficiency
***f***	Define a production function f(.) to represent how the input vector x is transformed into output vector **y**, e.g.: **y** = f (**x**).	Production function f(.)

### Variations of the baseline scenario

Our simulation scenarios included:

#### Varied sample sizes (a)

Sample size, or the number of DMUs under analysis, is an important factor that can affect the performance of DEA and rSDF-CD. For DEA, the model’s discriminatory power, or its ability to identify inefficient facilities, is largely defined by the number of inputs and outputs included in the model relative to the number of DMUs [40]. The issue of sample size is related to flexible weights, as having a larger number of inputs and outputs for a given sample size increases the likelihood of having a DMU with a particular ratio of outputs to inputs and no peers for comparison. Eventually, these DMUs may be scored as fully efficient. rSDF-CD is a regression-based approach, resulting in similar sample size requirements. While previous simulation studies have found that sample sizes less than 50 can be problematic for SDF [41], more recent work has shown that SDF can be appropriately used in settings with samples size smaller than 50 [33]. For our simulation study, we tested four different sample sizes: *n* = 20, *n* = 100, *n* = 200, and *n* = 1,000 (Table 1).

#### Varied correlations between inputs (b)

Correlation between inputs also need to be considered in comparing efficiency methods. For the present study, the following correlations were simulated: 0.60 between *x*_1_ and *x*_2_, 0.40 between *x*_1_ and *x*_3_, and 0.10 between *x*_2_ and *x*_3_. To generate uniformly distributed and correlated variables, we used an approach described elsewhere [42].

#### Varied fixed inputs (c)

Health service production is likely to require a minimum number of inputs, independently of the volume of services provided. The facility itself, where patients can be seen, is an example of a fixed input. To reflect this reality, we modeled each input with a fixed and variable component, as presented in Eq (9):

$$Y_{i}=((x_{1,\text{i}}-0.5)\cdot 0.2+(x_{2,\text{i}}-2.0)\cdot 0.5+(x_{3,\text{i}}-0.1)\cdot 0.3)$$ (9)

This is equivalent to a single fixed input equal to 0.5⋅0.2 + 2⋅0.5 + 0.1⋅0.3 and can be rewritten using a single constant term. To ensure that inputs take a value equal to or greater than zero after detraction of the fixed component, we increased the minimum value for each input by the respective fixed input. Inputs were drawn from the following distributions: *x*_1_ ∼ *unif*(0.5, 5), *x*_2_ ∼ *unif*(2.0, 10), and *x*_3_ ∼ *unif*(0.1, 8).

#### Varied measurement error (d)

Facility data are often noisy, so it is critical to include measurement error in sensitivity analyses. In this scenario, we included two types of error: additive and multiplicative error, which were both normally distributed, $v_{i}\sim N\left(0,\sigma_{v}^{2}\right)$ (Table B in S1 Appendix). For the additive error scenario, measurement error was added to each input and output. To avoid negative values for inputs and outputs that would prevent DEA from computing efficiency scores, we replaced any negative value with a small positive number (0.01). For the multiplicative error scenario, each input and output was scaled by the exponential of the measurement error. For each type of measurement error, we modeled three scenarios (Table C in S1 Appendix): (1) low measurement error (*σ*_*v*_ = 0.02); (2) high measurement error (*σ*_*v*_ = 0.08); and (3) mixed measurement error. For the latter, we assumed that relatively few DMUs (15%) were characterized by high measurement error (*σ*_*v*_ = 0.08), while most DMUs (85%) were characterized by low measurement error (*σ*_*v*_ = 0.02). DMUs were assigned low or high measurement error based on a random number, *τ*_*i*_ ∼ *unif*(0,1). This scenario reflected settings where data quality may vary substantially. We applied measurement error to all inputs and outputs. The total output capacity was calculated as described above with uniform distributed efficiency (see S1 Appendix for additional details).

#### Varied efficiency distribution (e)

Past studies have assumed positively-skewed distributions of efficiency [4–11], frequently applying half-normal, exponential, and gamma distributions. By definition, these distributions assume that most DMUs are efficient and only a small portion of DMUs would qualify as inefficient. We replicated our baseline simulation design using a half-normal distribution of efficiency, $\theta_{i}∼\text{exp}\left\{-\left|N\left(0,\sigma_{u}^{2}\right)\right|\right\}$. Different levels of efficiency variation were captured across DMUs by including low (*σ*_*u*_ = 0.05) and high (*σ*_*u*_ = 0.20) standard deviations of the efficiency distribution. We used the negative of the exponential of the efficiency term to bound efficiency values between zero and one.

#### Varied functional form (f)

The functional form of the multiple-output production function is frequently debated in selecting data generation processes. The most commonly-used functional forms in efficiency simulation studies are Cobb-Douglas and piecewise Cobb-Douglas [7,43]. We replicated both processes in our simulation, assuming that all inputs were drawn from a uniform distribution between 1 and 15, x ∼ *unif*(1, 15) (details shown in S1 Appendix). Multiple-output productions functions assume that the transformation function is separable, such that outputs are separable from inputs. We modeled a Cobb-Douglas output aggregate, while the input aggregate was modeled as Cobb-Douglas and piecewise Cobb-Douglas. We followed an approach used in past studies to ensure that that all outputs followed a uniform distribution (details shown in S1 Appendix) [44].

#### Varied functional form, measurement error, and efficiency distribution (a traditional simulation design) (g)

This simulation replicated a scenario for health service production in higher-income countries, the most prevalent setting for past efficiency studies. We modeled a Cobb-Douglas multiple-output production function with multiplicative measurement error applied to outputs only, $y_{r,i}\cdot e^{v_{r,i}}$ with $v_{r,i}∼N\left(0,\sigma_{v}^{2}\right)$. Low and high measurement errors were defined as above (*σ*_*v*_ = 0.02 and *σ*_*v*_ = 0.08, respectively). We created two types of models with half-normally distributed efficiencies: (1) a model which included low standard deviation for the error term and efficiency component (*σ*_*v*_ = 0.02, *σ*_*u*_ = 0.05); and (2) a model characterized by high standard deviation for the error term and the efficiency component (*σ*_*v*_ = 0.08, *σ*_*u*_ = 0.20). The simulation scenario matched *a priori* assumptions pertaining to the distributions of measurement error and efficiency. In addition, the rSDF-CD functional form was correctly specified, which provided the best-case performance scenario for the rSDF-CD approach.

### Performance criteria

We identified five performance criteria to evaluate how well efficiency was measured. These criteria were used to select the percentiles for rDEA, as well as to compare the performance of efficiency measurement approaches (DEA, rDEA, rSDF-CD, and ENS) for each simulation scenario. Mean absolute deviation and average rank correlations between true and estimated efficiency values are the most commonly-used performance and benchmarking criteria in the efficiency literature [5,7,45,46]. We used the median absolute deviation (MAD) instead of the mean absolute deviation, as mean metrics are more sensitive to outliers [10], and we used Spearman’s rank correlations (*r*_*s*_) to measure relative changes in DMU rankings across scenarios. A limitation of these performance indicators is that they do not distinguish between the overestimation and underestimation of efficiency. To address this concern, we also included the percentage of DMUs with excessively underestimated (PU_20_) and overestimated (PO_20_) efficiency. Differing from previous studies [33], we considered efficiency to be excessively underestimated or overestimated if the measured deviation between true and estimated efficiency levels exceeded 20 percentage points. By applying this threshold, we identified instances of overestimation and underestimation that could substantially affect a facility’s efficiency score. This classification can be particularly useful when the goal is to classify DMUs by four or five classes of efficiency levels (e.g., very low to very high efficiency), as changes exceeding 20 percentage points would likely reclassify DMUs at different levels of efficiency. Lastly, we included an indicator to identify the percentage of facilities that received an efficiency score of one when their true efficiency fell below 0.80 (NOTFront), capturing DMUs that were not actually at the efficiency frontier. This performance indicator was considered particularly important because the misidentification of fully-efficient facilities affects the estimated capacities of facility-level service production. For each of these performance criteria, we reported the average value over 2,000 replications.

## Results

We present our results in accordance with descriptions of each simulation scenario, starting with the novel simulation design for LMICs (baseline scenario) and moving through each scenario variation.

### Percentiles for rDEA and LMICs simulation design

Table 2 details the results from testing different percentiles for relative weight restrictions in rDEA. Imposing restrictions on DEA improved all performance criteria, even when the percentiles were relatively broad (20–80). rDEA performance generally improved as percentiles narrowed, though rates of underestimation (PU_20%_) increased when percentiles were narrower than 35–65, a point at which a trade-off emerged between the underestimation and overestimation of efficiency (PU_20%_ increased while PO_20%_ decreased) and MAD rose as well. When equal weights were applied to all DMUs, which corresponded with the median percentile for weight ratio distributions (50–50), model performance deteriorated across all criteria. These results indicated that imposing some degree of weighting flexibility is desirable, but overly-narrow restrictions may be detrimental. Based on these data, we set lower- and upper-bound restrictions to equal the 40–60 percentiles of their distributions. These restrictions minimized MAD; produced a *r*_*s*_ = 0.955; reduced NOTFront to 2.7% and overestimation to 7.2%; and kept underestimation low (1.5%).

**Table 2:** Performance of rDEA across different weight restriction percentile cutoffs.

Percentile	MAD	NOTFront	PU_20%_	PO_20%_	*r*_*s*_
0–0 (DEA)	0.065	10.9%	**0.0%**	41.4%	0.877
20–80	0.036	4.0%	**0.0%**	23.7%	0.949
25–75	0.031	3.4%	**0.0%**	19.0%	0.953
30–70	0.027	3.1%	0.1%	14.6%	0.955
35–65	**0.025**	2.8%	0.3%	10.6%	**0.956**
40–60	**0.025**	**2.7%**	1.5%	7.2%	0.955
45–55	0.030	**2.7%**	5.1%	4.8%	0.953
50–50	0.045	**2.7%**	16.1%	**3.6%**	0.949

Note: Numbers in **bold** highlight the best outcome for each performance indicator across the alternative approaches. MAD: median absolute deviation, NOTFront: percentage of misclassified DMUs, *PU*_20%_: percentage of underestimation, *PO*_20%_: percentage of overestimation, *r*_*s*_: Spearman’s rank correlation.

**Fig 1:**
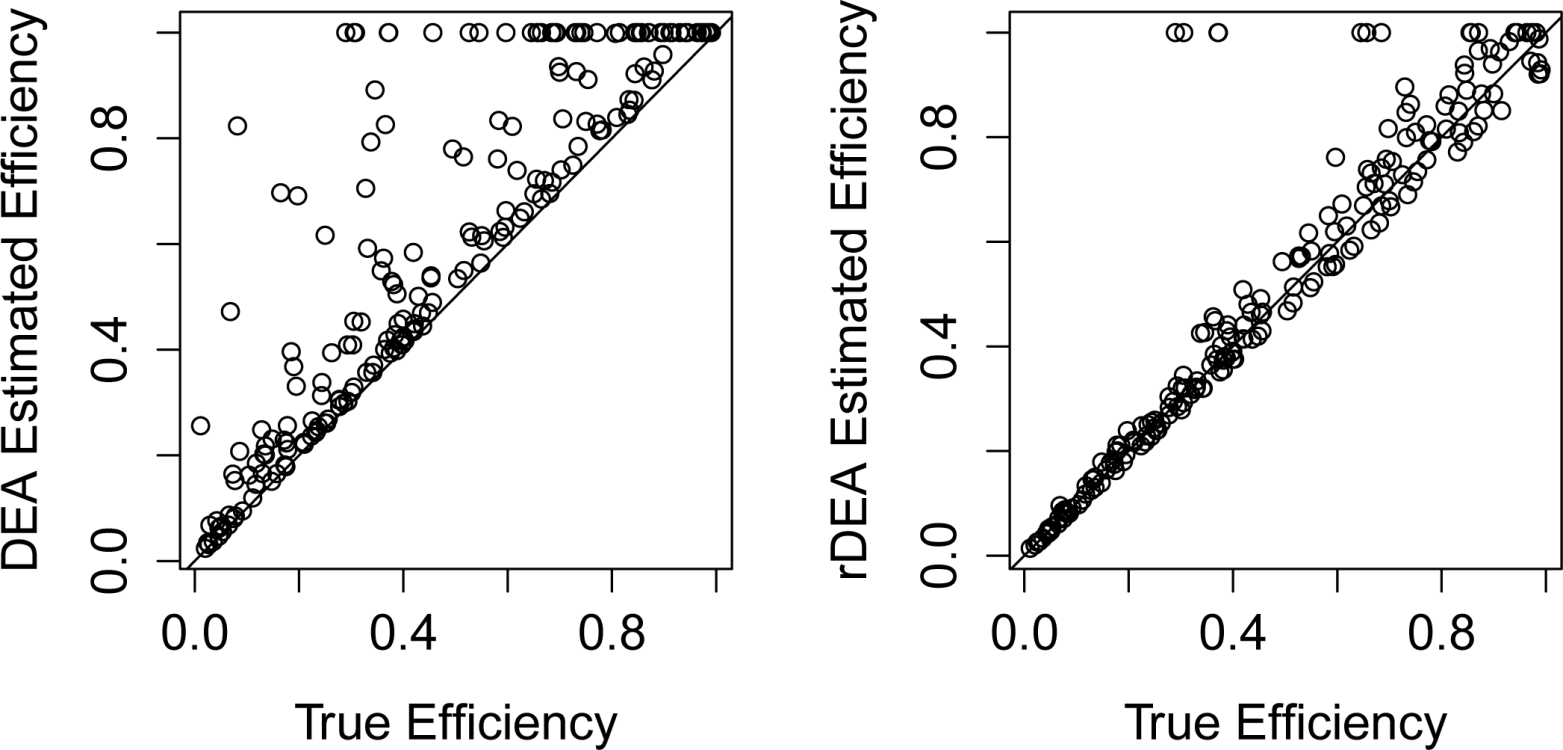
Comparison of DEA and rDEA estimated efficiency vs. true efficiency.

We then compared the performance of all efficiency estimation approaches for the LMIC scenario (baseline scenario) using the 40–60 percentiles as restrictions for rDEA (Table 3). DEA exhibited vastly superior performance to rSDF-CD, while rDEA provided even greater improvements upon DEA results. DEA resulted in efficiency overestimation for 42.6% of the sample and 11.8% misclassification. rDEA corrected for these issues, and reduced overestimation to 7.2%. Fig 1 shows these findings for one of the 2,000 replications, plotting the relationships between true and predicted levels of efficiency for DEA and rDEA. In general, a large proportion of facilities that were initially assigned to the efficiency frontier with DEA had their efficiency scores recalibrated to levels closer to true values with rDEA. Our ENS approach yielded a MAD similar to DEA, a very high Spearman’s rank correlation, and similar levels for PU_20%_ and PO_20%_.

**Table 3:** Performance for the LMIC setting (baseline simulation).

Method	MAD	NOTFront	PU_20%_	PO_20%_	*r*_*s*_
DEA	0.068	11.8%	**0.0%**	42.6%	0.863
rDEA	**0.025**	2.7%	1.5%	7.2%	**0.955**
rSDF-CD	0.106	**0.0%**	50.2%	10.5%	0.762
ENS	0.055	**0.0%**	25.8%	**6.3%**	0.936

Note: Numbers in **bold** highlight the best outcome for each performance indicator across the alternative approaches. MAD: median absolute deviation, NOTFront: percentage of misclassified DMUs, *PU*_20%_: percentage of underestimation, *PO*_20%_: percentage of overestimation, *r*_*s*_: Spearman’s rank correlation.

### Sample size (a)

Table 4 contains results from the scenario testing different sample sizes, with a particular focus on datasets with smaller samples (*n =* 20 to 100 DMUs) to reflect likely data scenarios for LMICs. All approaches were affected by very small datasets (*n* = 20), including rSDF-CD. DEA and rDEA experienced improving performance with increasing sample sizes, while rSDF-CD and ENS approaches were less sensitive to samples exceeding 20 DMUs. DEA-based methods generally improved with larger sample sizes; however, DEA resulted in high levels of efficiency overestimation in the scenario with 1,000 DMUs (PO_20%DEA_ = 20.5%). For smaller samples (*n =* 20), rDEA underestimated efficiency (PU_20%_ = 27.5%), but with larger sample sizes (*n ≥* 100), underestimated efficiency decreased to 6.1%. In scenarios with very large samples of DMUs (*n* = 1,000), rDEA estimates of efficiency nearly matched true efficiency. rSDF-CD performed better than DEA in terms of MAD and Spearman’s rank correlation with small sample sizes (*n* = 20). rDEA yielded the best performance for MAD with larger sample sizes (*n ≥* 100). Our ENS model performed similarly to rDEA for smaller sample sizes (*n =* 20), and maintained high performance in scenarios with larger sample sizes.

**Table 4:** Performance across variations in sample size.

Sample size	Method	MAD	NOTFront	PU_20%_	PO_20%_	*r*_*s*_
***n =* 20**	DEA	0.241	36.4%	**0.0%**	80.0%	0.747
	rDEA	0.123	7.5%	27.5%	**33.0%**	0.782
	rSDF-CD	0.149	**0.0%**	27.9%	40.3%	0.656
	ENS	**0.102**	**0.0%**	20.7%	34.7%	**0.793**
***n =* 100**	DEA	0.105	16.4%	**0.0%**	55.4%	0.837
	rDEA	**0.039**	3.3%	6.1%	13.8%	**0.936**
	rSDF-CD	0.111	**0.0%**	49.3%	13.3%	0.748
	ENS	0.059	**0.0%**	26.4%	**9.1%**	0.921
***n =* 200**	DEA	0.068	11.8%	**0.0%**	42.6%	0.863
	rDEA	**0.025**	2.7%	1.5%	7.2%	**0.955**
	rSDF-CD	0.106	**0.0%**	50.2%	10.5%	0.762
	ENS	0.055	**0.0%**	25.8%	**6.3%**	0.936
***n =* 1,000**	DEA	0.017	5.8%	**0.0%**	20.5%	0.907
	rDEA	**0.007**	2.6%	**0.0%**	**2.8%**	**0.963**
	rSDF-CD	0.099	**0.0%**	47.2%	10.2%	0.774
	ENS	0.051	**0.0%**	23.7%	5.5%	0.938

Note: Numbers in **bold** highlight the best outcome for each performance indicator across the alternative approaches. MAD: median absolute deviation, NOTFront: percentage of misclassified DMUs, *PU*_20%_: percentage of underestimation, *PO*_20%_: percentage of overestimation, *r*_*s*_: Spearman’s rank correlation.

### Correlated inputs (b)

Table 5 shows that correlations between inputs did not substantially change model performance, as all indicators remained largely unaffected by the inclusion of correlation in inputs.

**Table 5:** Performance across variations in the correlation structure between inputs.

Structure	Method	MAD	NOTFront	PU_20%_	PO_20%_	*r*_*s*_
**No correlation**	DEA	0.068	11.8%	**0.0%**	42.6%	0.863
	rDEA	**0.025**	2.7%	1.5%	7.2%	**0.955**
	rSDF-CD	0.106	**0.0%**	50.2%	10.5%	0.762
	ENS	0.055	**0.0%**	25.8%	**6.3%**	0.936
**Correlated inputs**	DEA	0.062	10.4%	**0.0%**	39.4%	0.874
	rDEA	**0.025**	2.8%	1.4%	7.3%	**0.954**
	rSDF-CD	0.103	**0.0%**	48.6%	10.6%	0.768
	ENS	0.054	**0.0%**	24.2%	**6.3%**	0.936

Note: Numbers in **bold** highlight the best outcome for each performance indicator across the alternative approaches. MAD: median absolute deviation, NOTFront: percentage of misclassified DMUs, *PU*_20%_: percentage of underestimation, *PO*_20%_: percentage of overestimation, *r*_*s*_: Spearman’s rank correlation.

### Fixed inputs (c)

Variations in fixed inputs had a limited effect on model performance (Table 6), and only rDEA was substantially affected by this change. Under this scenario, MAD for rDEA increased from 0.025 to 0.047, and the percentage of facilities with underestimated efficiency increased by 17.4 percentage points. The ENS approach was the least affected by the inclusion of fixed inputs.

**Table 6:** Performance across variation in fixed inputs.

Inputs	Method	MAD	NOTFront	PU_20%_	PO_20%_	*r*_*s*_
**No fixed inputs**	DEA	0.068	11.8%	**0.0%**	42.6%	0.863
	rDEA	**0.025**	2.7%	1.5%	7.2%	**0.955**
	rSDF-CD	0.106	**0.0%**	50.2%	10.5%	0.762
	ENS	0.055	**0.0%**	25.8%	**6.3%**	0.936
**With fixed inputs**	DEA	0.075	8.9%	**2.7%**	41.5%	0.887
	rDEA	**0.047**	2.2%	18.9%	8.9%	**0.930**
	rSDF-CD	0.099	**0.0%**	48.0%	9.7%	0.774
	ENS	0.061	**0.0%**	32.7%	**4.8%**	0.928

Note: Numbers in **bold** highlight the best outcome for each performance indicator across the alternative approaches. MAD: median absolute deviation, NOTFront: percentage of misclassified DMUs, *PU*_20%_: percentage of underestimation, *PO*_20%_: percentage of overestimation, *r*_*s*_: Spearman’s rank correlation.

### Measurement error (d)

Under scenarios where efficiency was uniformly distributed and different types of measurement error were added to inputs and outputs, results remained similar to our previous simulation scenarios (Table 7). In particular, rDEA performed better than rSDF-CD and DEA for MAD, percentage of DMUs with overestimated efficiency, and Spearman’s rank correlation. In terms of measurement error type, rDEA was less successful in reducing absolute overestimation when the measurement error was additive (PO_20%,rDEA_ = 9.8–15.3%) as compared with multiplicative error (PO_20%,rDEA_ = 7.4–9.3%) and mixed measurement error (PO_20%,rDEA_ = 8.0%). As expected, all performance criteria slightly declined when the variance of the measurement error was high (rather than low). Model performance pertaining to measurement error resulted in two main findings. First, rDEA appeared to be robust to measurement error in the data. The ENS approach was generally robust to the introduction of measurement error; however, when measurement error was additive, ENS often overestimated efficiency, akin to rSDF-CD and rDEA.

**Table 7:** Performance across variations in measurement error.

Type of error	Model specification	Method	MAD	NOTFront	PU_20%_	PO_20%_	*r*_*s*_
**Additive measurement error**	*θ*_*i*_ ∼ unif(0,1), v_i_ ∼ N(0, 0.02^2^)	DEA	0.069	11.8%	**0.1%**	44.2%	0.862
		rDEA	**0.022**	2.7%	0.2%	9.8%	**0.963**
		rSDF-CD	0.095	**0.0%**	32.2%	26.4%	0.797
		ENS	0.048	**0.0%**	9.6%	20.6%	0.940
	*θ*_*i*_ ∼ unif(0,1), v_i_ ∼ N(0, 0.08^2^)	DEA	0.072	12.0%	**0.8%**	45.7%	0.851
		rDEA	**0.028**	2.8%	1.8%	**15.3%**	**0.953**
		rSDF-CD	0.102	**0.0%**	35.0%	25.5%	0.779
		ENS	0.054	**0.0%**	13.8%	21.1%	0.929
**Multiplicative measurement error**	*θ*_*i*_ ∼ unif(0,1), v_i_ ∼ N(0, 0.02^2^)	DEA	0.068	11.8%	**0.0%**	42.6%	0.862
		rDEA	**0.026**	2.7%	2.0%	7.4%	**0.953**
		rSDF-CD	0.106	**0.0%**	50.3%	10.5%	0.762
		ENS	0.056	**0.0%**	26.5%	**6.3%**	0.935
	*θ*_*i*_ ∼ unif(0,1), v_i_ ∼ N(0, 0.08^2^)	DEA	0.071	11.9%	**0.4%**	42.7%	0.847
		rDEA	**0.043**	2.8%	12.4%	9.3%	**0.936**
		rSDF-CD	0.109	**0.0%**	51.1%	10.9%	0.759
		ENS	0.066	**0.0%**	34.1%	**6.5%**	0.926
**Mixed measurement error**	*θ*_*i*_ ∼ unif(0,1)	DEA	0.068	11.8%	**0.0%**	42.7%	0.859
		rDEA	**0.030**	2.8%	3.3%	8.0%	**0.950**
		rSDF-CD	0.107	**0.0%**	50.4%	10.6%	0.762
		ENS	0.058	**0.0%**	28.1%	**6.3%**	0.933

Note: Numbers in **bold** highlight the best outcome for each performance indicator across the alternative approaches. MAD: median absolute deviation, NOTFront: percentage of misclassified DMUs, *PU*_20%_: percentage of underestimation, *PO*_20%_: percentage of overestimation, *r*_*s*_: Spearman’s rank correlation.

### Efficiency distribution (e)

Across scenarios, rDEA performed better than DEA and rSDF-CD when efficiency followed a half-normal distribution (Table 8). By contrast, rSDF-CD performance substantially deteriorated under this scenario, leading to high levels of underestimation (PU_20%, rSDF−CD_ = 70.3%–82.0%). Minimal variations in efficiency often made it more challenging for rSDF-CD to accurately estimate efficiency. DEA performed relatively well, although its performance for Spearman’s rank correlation worsened when the efficiency distribution was half-normal, decreasing from *r*_*s*_ = 0.863 to *r*_*s*,*DEA*_ = 0.592–0.664. rDEA was robust to changes in efficiency distributions and variations in efficiency, generating estimates that aligned closely with true values, both when variation in efficiency was low (MAD_rDEA_ = 0.004, *r*_*s*,*rDEA*_ = 0.892) and high (MAD_rDEA_ = 0.012, *r*_*s*,*rDEA*_ = 0.905).rDEA’s overestimation and misclassification percentages were very low across all efficiency variation scenarios. ENS performed better than rSDF-CD in terms of MAD and Spearman’s rank correlations when efficiency variation was high, but still showed suboptimal performance in absolute terms when efficiency variation was low; this result was largely driven by low rSDF-CD performance. Overall, varying assumptions about efficiency distributions led to different concerns about modeling approaches. rDEA generally had the most robust performance across scenarios for efficiency distribution and variation. These results may be less informative than the findings from other simulation, as the combination of a linear efficiency frontier and half-normally distributed efficiency is unlikely to occur outside of simulation environments.

**Table 8:** Performance across variations in the efficiency distribution.

Efficiency distribution	Model specification	Method	MAD	NOTFront	PU_20%_	PO_20%_	*r*_*s*_
**Uniformly distributed efficiency**	*θ*_*i*_ ∼ unif(0,1)	DEA	0.068	11.8%	**0.0%**	42.6%	0.863
		rDEA	**0.025**	2.7%	1.5%	7.2%	**0.955**
		rSDF-CD	0.106	**0.0%**	50.2%	10.5%	0.762
		ENS	0.055	**0.0%**	25.8%	**6.3%**	0.936
**Half-normally distributed efficiency with low variation**	$\theta_{i}∼e^{{\text{N}}^{+}\left(0,0.05^{2}\right)}$	DEA	0.008	**0.0%**	**0.0%**	**0.0%**	0.592
		rDEA	**0.004**	**0.0%**	**0.0%**	**0.0%**	**0.892**
		rSDF-CD	0.367	**0.0%**	82.0%	**0.0%**	0.064
		ENS	0.184	**0.0%**	46.6%	**0.0%**	0.180
**Half-normally distributed efficiency with high variation**	$\theta_{i}∼e^{{\text{N}}^{+}\left(0,0.20^{2}\right)}$	DEA	0.028	4.5%	**0.0%**	8.8%	0.664
		rDEA	**0.012**	1.0%	**0.0%**	1.4%	**0.905**
		rSDF-CD	0.271	**0.0%**	70.3%	0.5%	0.238
		ENS	0.137	**0.0%**	36.7%	**0.3%**	0.540

Note: Numbers in **bold** highlight the best outcome for each performance indicator across the alternative approaches. MAD: median absolute deviation, NOTFront: percentage of misclassified DMUs, *PU*_20%_: percentage of underestimation, *PO*_20%_: percentage of overestimation, *r*_*s*_: Spearman’s rank correlation.

### Functional form (f)

While maintaining a uniform efficiency distribution and excluding measurement error, we tested different forms of the multiple-output production function, including Cobb-Douglas and piecewise Cobb-Douglas. rSDF-CD performed best using Cobb-Douglas and piecewise Cobb-Douglas multiple-output production functions, which was not surprising given that the model was correctly specified for rSDF-CD. For the Cobb-Douglas and piecewise Cobb-Douglas functional forms, MAD_rSDF−CD_ (0.012) was substantially lower than MAD_DEA_ (0.087–0.091) and MAD_rDEA_ (0.092–0.095). In comparison with DEA, rDEA did not show marked improvements for MAD but it reduced the percentage of DMUs misclassified (from a NOTFront of 11.6% to 3.1% with Cobb-Douglas and 9.5% to 2.9% with piecewise Cobb-Douglas) and increased Spearman’s rank correlation (in Cobb-Douglas from *r*_*s*,*rDEA*_ = 0.839 to *r*_*s*,*rDEA*_ = 0.874; in piecewise Cobb-Douglas from *r*_*s*,*rDEA*_ = 0.836 to *r*_*s*,*rDEA*_ = 0.891). rDEA was also successful in reducing efficiency overestimation, with DEA’s NOTFront equaling 42.0% and rDEA’s NOTFront equaling 12.6% with Cobb-Douglas; however, rDEA led to higher rates of efficiency underestimation across scenarios. Overall the results presented in Table 9 indicate that functional form was a primary determinant of most models’ performances. The performance of ENS for both Cobb-Douglas and piecewise Cobb-Douglas specifications rivaled the performance of the linear specification. For non-linear multiple-output production functions, ENS had a protective effect against efficiency underestimation as compared to rDEA (e.g., ENS *PU*_20%_ = 12.6% vs rDEA *PU*_20%_ = 45.4% with Cobb-Douglas).

**Table 9:** Performance across variations in the functional form.

Functional form	Model specification	Method	MAD	NOTFront	PU_20%_	PO_20%_	*r*_*s*_
**Linear**	*θ*_*i*_ ∼ unif(0,1)	DEA	0.068	11.8%	**0.0%**	42.6%	0.863
		rDEA	**0.025**	2.7%	1.5%	7.2%	**0.955**
		rSDF-CD	0.106	**0.0%**	50.2%	10.5%	0.762
		ENS	0.055	**0.0%**	25.8%	6.3%	0.936
**Cobb-Douglas**	*θ*_*i*_ ∼ unif(0,1)	DEA	0.087	11.6%	7.2%	42.0%	0.839
		rDEA	0.095	3.1%	45.4%	12.6%	0.874
		rSDF-CD	**0.012**	**0.0%**	**0.0%**	**0.2%**	**0.997**
		ENS	0.045	**0.0%**	12.6%	7.2%	0.960
**Piecewise Cobb-Douglas**	*θ*_*i*_ ∼ unif(0,1)	DEA	0.091	9.5%	16.5%	36.3%	0.836
		rDEA	0.092	2.9%	43.9%	13.3%	0.891
		rSDF-CD	**0.012**	**0.0%**	**0.0%**	**0.3%**	**0.997**
		ENS	0.044	**0.0%**	7.7%	7.5%	0.964

Note: Numbers in **bold** highlight the best outcome for each performance indicator across the alternative approaches. MAD: median absolute deviation, NOTFront: percentage of misclassified DMUs, *PU*_20%_: percentage of underestimation, *PO*_20%_: percentage of overestimation, *r*_*s*_: Spearman’s rank correlation.

### A traditional simulation design (g)

Table 10 shows the results for a scenario that mirrored health service production in higher-income settings (traditional data generation process), assuming a Cobb-Douglas multiple-output production function, presence of normally distributed measurement error, and half-normally distributed efficiency. These results were compared with those from a scenario in which efficiency was uniformly distributed and all other parameters were unchanged. rSDF-CD estimates of efficiency nearly matched true efficiency, independently of the efficiency distribution. These findings were expected for half-normally distributed efficiency, as the model was correctly specified and all assumptions were met. Notably, rSDF-CD remained robust when tested beyond a traditional efficiency distribution (uniform instead of half-normal). By contrast, DEA and rDEA performance deteriorated across scenarios, with high MADs, high levels of efficiency underestimation, and low Spearman’s rank correlations. ENS had similar MAD and Spearman’s rank correlations to those of DEA and rDEA, but its absolute performance remained unsatisfactory when efficiency was uniform, resulting in overestimation and misclassification of DMUs. When efficiency was uniformly distributed, rDEA successfully reduced overestimation to 12.6% (in comparison to 41.9% with DEA), but underestimation increased, rising to 45.7% (in comparison to 7.5% with DEA). ENS generally performed well in simulations with uniform efficiency (MAD_ENS_ = 0.047–0.065, *r*_*s*,*ENS*_ = 0.935–0.958), but performed poorly on the less likely scenario of half-normal efficiency (MAD_ENS_ = 0.158–0.207, *r*_*s*,*ENS*_ = 0.232–0.587), largely due to rDEA’s poor performance. Overall, DEA-based approaches estimated efficiency more accurately for uniform distributions than for half-normal distributions.

**Table 10:** Performance across variations in functional form, efficiency distribution, and measurement error.

Efficiency distribution	Model specification	Method	MAD	NOTFront	PU_20%_	PO_20%_	*r*_*s*_
**Half-normally distributed efficiency**	$\theta_{i}∼e^{{\text{N}}^{+}\left(0,0.05^{2}\right)},\;{\text{v}}_{\text{i}}∼\text{N}\left(0,0.02^{2}\right)$	DEA	0.198	**0.0%**	50.5%	**0.0%**	0.086
		rDEA	0.415	**0.0%**	83.1%	**0.0%**	0.098
		rSDF-CD	**0.008**	**0.0%**	**0.0%**	**0.0%**	**0.890**
		ENS	0.207	**0.0%**	56.7%	**0.0%**	0.232
	$\theta_{i}∼e^{{\text{N}}^{+}\left(0,0.20^{2}\right)},\;{\text{v}}_{\text{i}}∼\text{N}\left(0,0.08^{2}\right)$	DEA	0.162	4.6%	39.5%	8.7%	0.303
		rDEA	0.319	1.1%	76.9%	2.2%	0.338
		rSDF-CD	**0.027**	**0.0%**	**0.0%**	**0.0%**	**0.890**
		ENS	0.158	**0.0%**	43.9%	0.5%	0.587
**Uniformly distributed efficiency**	*θ*_*i*_ ∼ unif(0,1), v_i_ ∼ N(0, 0.02^2^)	DEA	0.087	11.6%	7.5%	41.9%	0.838
		rDEA	0.096	3.1%	45.7%	12.6%	0.873
		rSDF-CD	**0.016**	**0.0%**	**0.1%**	**0.6%**	**0.993**
		ENS	0.047	**0.0%**	14.9%	7.1%	0.958
	*θ*_*i*_ ∼ unif(0,1), v_i_ ∼ N(0, 0.08^2^)	DEA	0.095	11.5%	12.5%	40.8%	0.824
		rDEA	0.105	3.1%	50.4%	12.5%	0.858
		rSDF-CD	**0.044**	**0.0%**	**17.2%**	**4.3%**	**0.964**
		ENS	0.065	**0.0%**	34.1%	7.2%	0.935

Note: Numbers in **bold** highlight the best outcome for each performance indicator across the alternative approaches. MAD: median absolute deviation, NOTFront: percentage of misclassified DMUs, *PU*_20%_: percentage of underestimation, *PO*_20%_: percentage of overestimation, *r*_*s*_: Spearman’s rank correlation.

## Discussion

This study is, to our knowledge, the first-ever to empirically test the performance of efficiency measurement methods in a simulation environment specifically designed to reflect health service production in LMICs. We modified the data generation process commonly used in traditional simulation studies and assessed the performance of two well-established approaches for efficiency measurement, DEA and rSDF-CD. We also included an easy-to-implement weight restriction approach for DEA (rDEA), which offers a solution to using arbitrary weights in the absence of market information regarding relative weights, and combined rDEA with rSDF-CD to develop an ensemble model, ENS.

We found that when assumptions regarding efficiency distribution and functional form were adjusted to reflect LMIC settings, the accuracy of DEA and rSDF-CD methods in estimating efficiency declined. In our study, functional form of the multiple-output production function was the main determinant of model performance. We found that rSDF-CD was the preferred approach for Cobb-Douglas or piecewise Cobb-Douglas multiple-output production functions; however, when the multiple-output production function was linear, rDEA performed the best.

A main challenge in analyzing efficiency is being able to identify the underlying multiple-output production function and then selecting the most appropriate measurement approach. Tests such as the likelihood ratio test are commonly used to determine the preferred functional form for nested models (e.g., Cobb-Douglas versus translog functional form) [47]; however they cannot be used for comparing non-nested models, such as linear and Cobb-Douglas multiple-output production functions. We investigated the performance of a variety of functional form tests (S2 Appendix), and found them unreliable, indicating their potential limits for applied efficiency analyses. Further, these results point to the analytical issues that can arise when method choices are left to analysts.

Relatedly, we found that ENS, wherein efficiency estimates from rDEA and rSDF-CD were combined, provided the best solution for estimating efficiency in cases where the underlying production function is uncertain. ENS was robust across simulation designs for the linear production, Cobb-Douglas, and piecewise Cobb-Douglas with uniformly distributed efficiency. ENS also addressed one of rDEA’s largest pitfalls: its tendency to substantially underestimate efficiency in the presence of a non-linear multiple-output production function. Although ENS did not perform as well when efficiency was half-normally distributed and standard deviation low, this data scenario is unlikely to reflect the realities of health service production in LMICs.

We also found that DEA resulted in high levels of efficiency overestimation and misclassification of DMU efficiency when the efficiency distribution was uniform. These performance issues diminished when efficiency followed a half-normal distribution, emphasizing the importance of understanding how production levels are distributed across facilities when analyzing efficiency. rDEA and ENS were successful in addressing these limitations, suggesting that these approaches may be preferred for analyzing efficiency in lower-resource settings.

Estimating efficiency for multiple outputs requires greater model complexity and parameterization, which accounts for differences in results from our study and previous analyses [33]. When we replicated the findings for single-output production functions (Tables H and I in S3 Appendix), ENS performed well overall, including scenarios with half-normally distributed efficiency. Although estimating multiple output production processes is analytically challenging, it necessary to capture the realities of health service production, as very few, if any, health facilities produce only one output. We found that ENS may provide a viable estimation option for both single and multiple-output production functions, an important step toward improving the applications of efficiency analyses.

Additional work is needed to confirm the broader generalizability of our ENS approach, which may include testing more flexible forms of the efficiency frontier, such as the transcendental logarithmic (translog) form; analyzing a broader range of efficiency distributions, such as exponential or gamma distributions; studying different distributions for the inputs and outputs; and incorporating the performance of rSDF-CD under different misspecification issues.

Our findings have a number of applications, particularly as health policymakers and program leaders increasingly seek ways to heighten efficiency of health service production [48,49]. This is particularly relevant to LMICs, where improvements in health system access, demand for health care, and efforts to reach universal health coverage are resulting in growing patient volumes amid constrained budgets [38,50–52]. Through improved efficiency at the facility level, more patients can be diagnosed and treated without necessarily requiring a proportional increase in facility resources. Therefore there is a strong argument for routinely measuring and monitoring efficiency. Past studies have largely relied on traditional DEA models to assess technical efficiency [53–55], which, based on our study, may have resulted in the overestimation of efficiency in many settings. In these cases, facilities identified as “best performers” in service production may actually experience lower levels of efficiency and generate fewer services than previously estimated. By improving the accuracy of efficiency measurement in LMICs, we also move closer to empirically identifying determinants of heightened efficiency and developing data-driven policy interventions to improve the use of limited resources.

## Conclusions

This study provides new insights into efficiency measurement in low-resource settings through an innovative Monte Carlo simulation design. We developed a new data generation process for testing efficiency estimation methods for LMICs and compared the performance of established and novel approaches for measuring efficiency. We found that current efficiency estimation approaches are likely to overestimate efficiency levels and score individual facilities as fully efficient when their true performance is substantially lower. An ensemble model (ENS), consisting of averaging efficiency estimates drawn from a restricted version of DEA (rDEA) and restricted SDF-CD (rSDF-CD), performed most robustly across sensitivity analyses. In cases where the underlying multiple-output production function of a given dataset is uncertain, we recommend the use of ENS for analyzing efficiency. Although efficiency is one of many health system objectives, more accurate measurements of efficiency can provide an improved understanding of how health system performance and provision of health services can be maximized.

## Supporting Information

S1 Appendix Detailed description of the simulation design. This supplementary file provides additional information on the baseline simulation design and functional forms. Variations in functional form for a multiple-output production function are detailed in **Table A**. **Tables B and C** provide information how measurement error was varied as part of the simulation study.
(DOCX)

S2 Appendix Tests for functional form. This supplementary file details tests conducted to identify the true underlying functional forms across simulation scenarios. Testes include monotonicity violations (**Table D**), Ramsey RESET test (**Table E**), and minimization of RMSE (**Tables F and G**).
(DOCX)

S3 Appendix Results for the single-output production function. Results on functional form (**Table H**) and inefficiency distributions (**Table I**) are included in this supplementary file.
(DOCX)

## Abbreviations

ART: Antiretroviral therapy
CRS: Constant returns to scale
DEA: Data Envelopment Analysis
DMU: Decision-making unit
ENS: Ensemble model
GHDx: Global Health Data Exchange
LMICs: Low- and middle-income countries
MAD: Median absolute deviation
NOTFront: Not at the efficiency frontier
rDEA: Restricted Data Envelopment Analysis
SFA: Stochastic Frontier Analysis
SDF: Stochastic Distance Function, SDF
SDF-CD: Stochastic Distance Function with a Cobb-Douglass multiple-output production function
rSDG-CD: restricted Stochastic Distance Function with a Cobb-Douglass multiple-output production function
VRS: Variable returns to scale

## References

[1] Chisholm D, Evans DB. Improving health system efficiency as a means of moving towards universal coverage Geneva, Switzerland: World Health Organization (WHO); 2010 Available: http://www.who.int/entity/healthsystems/topics/financing/healthreport/28UCefficiency.pdf

[2] Bennett S, Ozawa S, Rao KD. Which Path to Universal Health Coverage? Perspectives on the World Health Report 2010. PLoS Med. 2010;7: e1001001 10.1371/journal.pmed.1001001 21124959 PMC2988776

[3] Mortimer D. Competing Methods for Efficiency Measurement: a Systematic Review of Direct DEA vs SFA/DFA Comparisons. East Anglia, UK: Centre for Health Program Evaluation; 2002. Available: http://www.buseco.monash.edu.au/centres/che/pubs/wp136.pdf

[4] Burton A, Altman DG, Royston P, Holder RL. The design of simulation studies in medical statistics. Stat Med. 2006;25: 4279–4292. 10.1002/sim.2673 16947139

[5] Andor M, Hesse F. The StoNED age: the departure into a new era of efficiency analysis? A monte carlo comparison of StoNED and the “oldies” (SFA and DEA). J Product Anal. 2013;41: 85–109. 10.1007/s11123-013-0354-y

[6] Ruggiero J. A new approach for technical efficiency estimation in multiple output production. Eur J Oper Res. 1998;111: 369–380. 10.1016/S0377-2217(97)00351-2

[7] Banker RD, Gadh VM, Gorr WL. A Monte Carlo comparison of two production frontier estimation methods: Corrected ordinary least squares and data envelopment analysis. Eur J Oper Res. 1993;67: 332–343. 10.1016/0377-2217(93)90289-Y

[8] Estelle SM, Johnson AL, Ruggiero J. Three-stage DEA models for incorporating exogenous inputs. Comput Oper Res. 2010;37: 1087–1090. 10.1016/j.cor.2009.09.015

[9] Yu C. The effects of exogenous variables in efficiency measurement—A monte carlo study. Eur J Oper Res. 1998;105: 569–580. 10.1016/S0377-2217(97)00076-3

[10] Banker RD, Chang H, Cooper WW. A simulation study of DEA and parametric frontier models in the presence of heteroscedasticity. Eur J Oper Res. 2004;153: 624–640. 10.1016/S0377-2217(02)00699-9

[11] Resti A. Efficiency measurement for multi-product industries: A comparison of classic and recent techniques based on simulated data. Eur J Oper Res. 2000;121: 559–578. 10.1016/S0377-2217(99)00054-5

[12] Chen W-C, Johnson AL. A unified model for detecting efficient and inefficient outliers in data envelopment analysis. Comput Oper Res. 2010;37: 417–425. 10.1016/j.cor.2009.06.010

[13] Kneip A, Simar L, Wilson PW. Asymptotics and Consistent Bootstraps for DEA Estimators in Nonparametric Frontier Models. Econom Theory. 2008;24: 1663–1697.

[14] Bifulco R, Bretschneider S. Estimating school efficiency: A comparison of methods using simulated data. Econ Educ Rev. 2001;20: 417–429. 10.1016/S0272-7757(00)00025-X

[15] Henningsen G, Henningsen A, Jensen U. A Monte Carlo study on multiple output stochastic frontiers: a comparison of two approaches. J Product Anal. 2014;44: 309–320. 10.1007/s11123-014-0416-9

[16] Cooper WW, Seiford LM, Zhu J, editors. Handbook on Data Envelopment Analysis. 2nd ed. 2011 edition. New York, NY: Springer; 2011.

[17] Charnes A, Cooper WW, Lewin AY, Seiford LM, editors. Data Envelopment Analysis: Theory, Methodology, and Applications 1994 edition. Boston: Springer; 1995.

[18] Grosskopf S, Valdmanis V. Measuring hospital performance. A non-parametric approach. J Health Econ. 1987;6: 89–107. 10312167 10.1016/0167-6296(87)90001-4

[19] Hernández AR, Sebastián MS. Assessing the technical efficiency of health posts in rural Guatemala: a data envelopment analysis. Glob Health Action. 2014;7 10.3402/gha.v7.23190 PMC3901389 24461356

[20] Marschall P, Flessa S. Efficiency of primary care in rural Burkina Faso. A two-stage DEA analysis. Health Econ Rev. 2011;1: 5 10.1186/2191-1991-1-5 22828358 PMC3395044

[21] Portela MCAS, Thanassoulis E. Zero weights and non-zero slacks: Different solutions to the same problem. Ann Oper Res. 2006;145: 129–147. 10.1007/s10479-006-0029-4

[22] Perelman S, Santin D. Measuring educational efficiency at student level with parametric stochastic distance functions: an application to Spanish PISA results. Educ Econ. 2011;19: 29–49. 10.1080/09645290802470475

[23] Irz X, Thirtle C. Dual Technological Development in Botswana Agriculture: A Stochastic Input Distance Function Approach. J Agric Econ. 2004;55: 455–478. 10.1111/j.1477-9552.2004.tb00110.x

[24] Coelli T, Perelman S. A comparison of parametric and non-parametric distance functions: With application to European railways. Eur J Oper Res. 1999;117: 326–339. 10.1016/S0377-2217(98)00271-9

[25] Coelli T. On the econometric estimation of the distance function representation of a production technology. Université catholique de Louvain, Center for Operations Research and Econometrics (CORE); 2000. Report No.: 2000042. Available: https://ideas.repec.org/p/cor/louvco/2000042.html

[26] Jacobs R, Smith PC, Street A. Measuring Efficiency in Health Care: Analytic Techniques and Health Policy Cambridge; New York: Cambridge University Press; 2006.

[27] Jondrow J, Knox Lovell CA, Materov IS, Schmidt P. On the estimation of technical inefficiency in the stochastic frontier production function model. J Econom. 1982;19: 233–238.

[28] Lovell CAK, Travers P, Richardson S, Wood L. Resources and Functionings: A New View of Inequality in Australia In: Eichhorn PDD h c W, editor. Models and Measurement of Welfare and Inequality. Springer Berlin Heidelberg; 1994 pp. 787–807. Available: http://link.springer.com/chapter/10.1007/978-3-642-79037-9_41

[29] Greene WH. A Gamma-distributed stochastic frontier model. J Econom. 1990;46: 141–163.

[30] Stevenson RE. Likelihood functions for generalized stochastic frontier estimation. J Econom. 1980;13: 57–66.

[31] Farsi M, Filippini M. An Analysis of Efficiency and Productivity in Swiss Hospitals. Swiss J Econ Stat SJES. 2006;142: 1–37.

[32] Griffin JE, Steel MFJ. Bayesian stochastic frontier analysis using WinBUGS. J Product Anal. 2007;27: 163–176. 10.1007/s11123-007-0033-y

[33] Andor M, Hesse F. A Monte Carlo simulation comparing DEA, SFA and two simple approaches to combine efficiency estimates. CAWM Discuss Pap. 2011; Available: http://ideas.repec.org/p/zbw/cawmdp/51.html

[34] Nyman J, Bricker DL. Profit Incentives and Technical Efficiency in the Production of Nursing Home Care. Rev Econ Stat. 1989;71: 586–94.

[35] Institute for Health Metrics and Evaluation (IHME). Access, Bottlenecks, Costs, and Equity (ABCE) Project | GHDx. [cited 11 Sep 2015]. Available: http://ghdx.healthdata.org/series/access-bottlenecks-costs-and-equity-abce-project

[36] Yaya Bocoum F, Kouanda S, Kouyaté B, Hounton S, Adam T. Exploring the effects of task shifting for HIV through a systems thinking lens: the case of Burkina Faso. BMC Public Health. 2013;13: 997 10.1186/1471-2458-13-997 24148691 PMC4016414

[37] Joshi R, Alim M, Kengne AP, Jan S, Maulik PK, Peiris D, et al Task Shifting for Non-Communicable Disease Management in Low and Middle Income Countries–A Systematic Review. PLoS ONE. 2014;9: e103754 10.1371/journal.pone.0103754 25121789 PMC4133198

[38] Institute for Health Metrics and Evaluation (IHME). Health Service Provision in Zambia: Assessing Facility Capacity, Costs of Care, and Patient Perspectives Seattle, WA: IHME; 2014.

[39] Otto PB and L. Benchmarking: Benchmark and Frontier Analysis Using DEA and SFA. 2015. Available: https://cran.r-project.org/web/packages/Benchmarking/index.html

[40] Avkiran NK. Productivity Analysis in the Service Sector with Data Envelopment Analysis. N.K. Avkiran; 2006.

[41] Ruggiero J. Efficiency estimation and error decomposition in the stochastic frontier model: A Monte Carlo analysis. Eur J Oper Res. 1999;115: 555–563. 10.1016/S0377-2217(98)00245-8

[42] Dias CT dos S, Samaranayaka A, Manly B. On the use of correlated beta random variables with animal population modelling. Ecol Model. 2008;215: 293–300. 10.1016/j.ecolmodel.2008.03.020

[43] Hadad Y, Friedman L, Rybalkin V, Sinuany-Stern Z. The relationship between DEA efficiency and the type of production function, the degree of homogeneity, and error variability. Cent Eur J Oper Res. 2012;21: 595–607. 10.1007/s10100-012-0249-4

[44] Collier T, Johnson AL, Ruggiero J. Technical efficiency estimation with multiple inputs and multiple outputs using regression analysis. Eur J Oper Res. 2011;208: 153–160. 10.1016/j.ejor.2010.08.024

[45] Ruggiero J. A comparison of DEA and the stochastic frontier model using panel data. Int Trans Oper Res. 2007;14: 259–266. 10.1111/j.1475-3995.2007.00585.x

[46] Banker RD, Charnes A, Cooper WW, Maindiratta A. A Comparison of DEA and Translog Estimates of Production Frontiers Using Simulated Observations from a Known Technology In: Dogramaci A, Färe R, editors. Applications of Modern Production Theory: Efficiency and Productivity. Springer Netherlands; 1988 pp. 33–55. Available: http://link.springer.com/chapter/10.1007/978-94-009-3253-1_2

[47] Kneller R, Andrew Stevens P. The specification of the aggregate production function in the presence of inefficiency. Econ Lett. 2003;81: 223–226. 10.1016/S0165-1765(03)00173-3

[48] Yip W, Hafez R. Reforms for improving the efficiency of health systems: lessons from 10 country cases Geneva, Switzerland: World Health Organization (WHO); 2015 Available: http://www.who.int/health_financing/topics/efficiency-cost-effectiveness/synthesis_report/en/

[49] World Health Organization (WHO). World Health Report 2010 Health Systems Financing: the Path to Universal Coverage. Geneva, Switzerland: WHO; 2010 Available: http://www.who.int/whr/2010/en/10.2471/BLT.10.078741 PMC2878164 20539847

[50] Bärnighausen T, Bloom DE, Humair S. Universal antiretroviral treatment: the challenge of human resources. Bull World Health Organ. 2010;88: 951–952. 10.1590/S0042-96862010001200018 21124722 PMC2995182

[51] Institute for Health Metrics and Evaluation (IHME). Health Service Provision in Kenya: Assessing Facility Capacity, Costs of Care, and Patient Perspectives Seattle, WA: IHME; 2014 Available: http://www.healthdata.org/policy-report/health-service-provision-kenya-assessing-facility-capacity-costs-care-and-patient

[52] Institute for Health Metrics and Evaluation (IHME). Health Service Provision in Uganda: Assessing Facility Capacity, Costs of Care, and Patient Perspectives Seattle, WA: IHME; 2014 Available: http://www.healthdata.org/policy-report/health-service-provision-uganda-assessing-facility-capacity-costs-care-and-patient

[53] Akazili J, Adjuik M, Chatio S, Kanyomse E, Hodgson A, Aikins M, et al What are the Technical and Allocative Efficiencies of Public Health Centres in Ghana? Ghana Med J. 2008;42: 149–155. 19452023 PMC2673839

[54] Yawe B. Hospital Performance Evaluation in Uganda: A Super-Efficiency Data Envelope Analysis Model. Zamb Soc Sci J. 2010;1 Available: http://scholarship.law.cornell.edu/zssj/vol1/iss1/6

[55] Zere E, Mbeeli T, Shangula K, Mandlhate C, Mutirua K, Tjivambi B, et al Technical efficiency of district hospitals: Evidence from Namibia using Data Envelopment Analysis. Cost Eff Resour Alloc. 2006;4: 5 10.1186/1478-7547-4-5 16566818 PMC1524815

