# Correction: Efficiency of Health Care Production in Low-Resource Settings: A Monte-Carlo Simulation to Compare the Performance of Data Envelopment Analysis, Stochastic Distance Functions, and an Ensemble Model

**DOI:** 10.1371/journal.pone.0150570

**Published:** 2016-02-25

**Authors:** Laura Di Giorgio, Abraham D. Flaxman, Mark W. Moses, Nancy Fullman, Michael Hanlon, Ruben O. Conner, Alexandra Wollum, Christopher J. L. Murray

The first author’s name is cited incorrectly. The correct name for the citation is: Di Giorgio L. The correct citation is: Di Giorgio L, Flaxman AD, Moses MW, Fullman N, Hanlon M, Conner RO, et al. (2016) Efficiency of Health Care Production in Low-Resource Settings: A Monte-Carlo Simulation to Compare the Performance of Data Envelopment Analysis, Stochastic Distance Functions, and an Ensemble Model. PLoS ONE 11(1): e0147261. doi:10.1371/journal.pone.0147261

The Copyright statement is inaccurate. The correct statement is: Copyright: (c) 2016 Di Giorgio et al.
