# Supplementary material for: Efficiency of Health Care Production in Low-Resource Settings: A Monte-Carlo Simulation to Compare the Performance of Data Envelopment Analysis, Stochastic Distance Functions, and an Ensemble Model
Source: PLoS One. 2016 Jan 26;11(1):e0147261. doi: 10.1371/journal.pone.0147261 (PMC4727806; doi:10.1371/journal.pone.0147261)
Supplement: S1 Appendix — This supplementary file provides additional information on the baseline simulation design and functional forms. Variations in functional form for a multiple-output production function are detailed in Table A. Tables B and C provide information how measurement error was varied as part of the simulation study. (DOCX) [file pone.0147261.s001.docx]

**S1 Appendix. Detailed description of the simulation design**

**Baseline simulation design**

We modeled a multi-input, multi-output production function as shown in equation (1). We assumed that the production technology can be represented by the transformation of three discretionary inputs, $x_{1}, x_{2},$ and $x_{3}$, into a total productive capacity $Y$, according to the following linear production function which satisfied constant returns to scale (CRS):

$Y_{i}={0.2 x}_{1,i}+0.5 x_{2,i}+0.3 x_{3,i}$ (1)

where $i$ is the facility-index. We also assumed that inefficient behavior exists and can be modeled with uniformly distributed efficiency$, u_{i} \sim unif\left( 0,1 \right)$. The efficiency score scaled down the total productive capacity, ($Y_{i}^{'}$), $Y_{i}^{'}= Y_{i}\cdot u_{i}$. Three inputs were drawn for a sample of 200 facilities from the following uniform distributions:$x_{1} \sim unif(0,5)$, $x_{2} \sim unif(0, 10)$, and $x_{3} \sim unif\left( 0, 8 \right).$ Total productive capacity was used to produce up to three outputs. In reality, however, it is unlikely that every decision-making unit (DMU) produces all possible outputs. We accounted for such scenarios with a model specification that assumed all facilities produce output$y_{3}$, while only a subset of facilities produces output$y_{1}$ and/or output$y_{2}$. Production of these two outputs was defined by a random parameter. Each facility was assigned two random numbers$,q_{ji} \sim unif (0, 1)$, one for each output of choice. The production rule was defined in equation (2):

$D_{1,i}=1 iff q_{1,i}<0.4$ (2)

$$D_{2,i}=1 iff q_{2,i}<0.6$$

$$D_{3,i}=1 \forall q_{3, i}.$$

A DMU produced output $y_{1}$ only if it was assigned a random number smaller than 0.4 ($D_{1,i}=1$). Similarly, DMU produced output $y_{2}$ only if it was assigned a random number smaller than 0.6 ($D_{2,i}=1$). For each positive output, we determined how much of the total output capacity was devoted to its production (output shares). Output shares ($s_{ji})$were defined exogenously to reflect that demand was given, and that the shares were drawn from a uniform distribution when $D_{j,i}=1$,

$$s_{ji} \sim\left\{ \begin{aligned} unif\left( 0,1 \right), if D_{j,i}=1; \\ 0, \mathrm{otherwise}. \end{aligned} \right.$$

To ensure that the sum of output shares equaled one, output-specific productive capacity ($Y_{ji}^{s}$) was calculated as shown in equation (3):

$Y_{ji}^{s}= {(s}_{ji}/\sum_{j=1,2,3} s_{ji})\cdot Y_{i}^{'}.$ (3)

Last, we assumed that each output’s volume ($y_{ji}^{\mathrm{obs}}$) was dependent on the resources required to produce the output. If a particular output was more resource-intensive to produce, given a DMU’s productive capacity, fewer outputs could be produced by the DMU. The final volume of outputs produced by a DMU was defined in equation (4), where we assumed that output $y_{3}$ was the most resource-intensive output to produce, followed by $y_{2}$ and $y_{1}$.

$y_{1,i}^{\mathrm{obs}}= Y_{1i}^{s}/0.25$ (4)

$$y_{2,i}^{\mathrm{obs}}= Y_{2i}^{s}/0.5$$

$y_{3,i}^{\mathrm{obs}}= Y_{3i}^{s}/1.0$.

**Functional form**

We replicated a Cobb-Douglas and piecewise Cobb-Douglas multiple-output production function assuming that all inputs were drawn from a uniform distribution between 1 and 15, $x \sim unif(1, 15)$ (Table A). Multiple-output productions functions assume that the transformation function is separable, such that outputs are separable from inputs. In this design we modeled the output aggregate as Cobb-Douglas, while the input aggregate was modeled as a Cobb-Douglas and piecewise Cobb-Douglas. To model the output aggregate, we followed the approach used by Collier and Ruggiero [1], which ensured that all outputs followed a uniform distribution. This approach was preferred for computing a Cobb-Douglas multiple-output production function, as other methods have led to high skewedness for one of the outputs [2].

Inputs were drawn from a uniform distribution, after which an input aggregate was constructed based on the form,$f\left( x \right)= {x_{1}}^{\beta_{1}}{x_{2}}^{\beta_{2}}{x_{3}}^{\beta_{3}}$, with $\beta_{1}=0.2, \beta_{2}=0.5$, and $\beta_{3}=0.3$. Further, inefficient behavior was modeled as $g(y)=f(x)\cdot e^{{-u}_{i}}$ where $u_{i}\sim N^{+}\left( 0, \sigma_{u}^{2} \right).$

To generate outputs, we used three normally distributed random variables,$h_{j}\sim N\left( 0,1 \right), for j=1,2,3$. We defined each output as $y_{j}=\frac{e^{h_{j}}}{z},$ where $z$ was chosen to satisfy the equation$g\left( y \right)={y_{1}}^{\alpha_{1}}{y_{2}}^{\alpha_{2}}{y_{3}}^{\alpha_{3}}$. The values of output exponents were specified analogously to the inputs, with$\alpha_{1}=0.2, \alpha_{2}=0.5$, and $\alpha_{3}=0.3$.

**Table A. Variations in the functional form of the multiple-output production function.**

| **Functional form** | **Multiple-output production function specification** | **Input coefficients parameterization** |
| --- | --- | --- |
| Cobb-Douglas | ${y_{1}}^{\alpha_{1}}{y_{2}}^{\alpha_{2}}{y_{3}}^{\alpha_{3}}={x_{1}}^{\beta_{1}}{x_{2}}^{\beta_{2}}{x_{3}}^{\beta_{3}}$ | $\beta_{1}=0.2$, $\beta_{2}=0.5$, $\beta_{3}=0.3$ |
| Piecewise Cobb-Douglas | ${y_{1}}^{\alpha_{1}}{y_{2}}^{\alpha_{2}}{y_{3}}^{\alpha_{3}}={x_{1}}^{\beta_{1}}{x_{2}}^{\beta_{2}}{x_{3}}^{\beta_{3}}$ | For $x_{1}$≤ 5, $x_{2}$≤ 5, $x_{3}$≤ 5  $\beta_{1}$= 0.2, $\beta_{2}$= 0.5, $\beta_{3}$= 0.3    For 5 <$x_{1}$ ≤ 10, 5 <$x_{2}$≤ 10, 5 <$x_{3}$≤ 10  $\beta_{1}$= 0.15, $\beta_{2}$= 0.45, $\beta_{3}$= 0.25  For 10 <$x_{1}$ ≤ 15, 10 <$x_{2}$≤ 15, 10 <$x_{3}$≤ 15  $\beta_{1}$= 0.1, $\beta_{2}$= 0.4, $\beta_{3}$= 0.2 |

**Varied measurement error (simulation scenario *g*)**

Tables B and C provide additional detail regarding variations implemented as part of the simulation scenario *g*.

**Table B. Measurement error types.**

| **Additive measurement error** | **Multiplicative error** |
| --- | --- |
| For $z_{i}+$ $v_{i}\geq0$: $z_{i}=z_{i}+$ $v_{i} \forall z_{i}$  For $z_{i}+$ $v_{i}<0$: $z_{i}=0.01 \forall z_{i}$  with $z_{i}=x_{\mathrm{ri}}, y_{\mathrm{ji}}$ | For all $z_{i}$: $z_{i}\cdot e^{v_{i}} \forall z_{i}$  with $z_{i}=x_{\mathrm{ri}}, y_{\mathrm{ji}}$ |

**Table C. Measurement error scenarios.**

| **Low measurement error** | **High measurement error** | **Mixed measurement error** |
| --- | --- | --- |
| $\sigma_{v}^{2}$= 0.02 | $\sigma_{v}^{2}$= 0.08 | $\tau_{i} \sim unif(0,1)$  For $\tau_{i}\leq0.15$: $\sigma_{v}^{2}$ = 0.08  For$\tau_{i}>0.15$: $\sigma_{v}^{2}$ = 0.02 |

**References**

1. Collier T, Johnson AL, Ruggiero J. Technical efficiency estimation with multiple inputs and multiple outputs using regression analysis. Eur J Oper Res. 2011;208: 153–160. doi:10.1016/j.ejor.2010.08.024

2. Bifulco R, Bretschneider S. Estimating school efficiency: A comparison of methods using simulated data. Econ Educ Rev. 2001;20: 417–429. doi:10.1016/S0272-7757(00)00025-X
