# Supplementary material for: Efficiency of Health Care Production in Low-Resource Settings: A Monte-Carlo Simulation to Compare the Performance of Data Envelopment Analysis, Stochastic Distance Functions, and an Ensemble Model
Source: PLoS One. 2016 Jan 26;11(1):e0147261. doi: 10.1371/journal.pone.0147261 (PMC4727806; doi:10.1371/journal.pone.0147261)
Supplement: S2 Appendix — This supplementary file details tests conducted to identify the true underlying functional forms across simulation scenarios. Testes include monotonicity violations (Table D), Ramsey RESET test (Table E), and minimization of RMSE (Tables F and G). (DOCX) [file pone.0147261.s002.docx]

**S2 Appendix. Tests for functional form**

Simulation scenario *f* showed that the underlying functional form was the primary determinant of model performance for Data Envelopment Analysis (DEA), restricted DEA (rDEA), and a restricted Stochastic Distance Function with an assumed Cobb-Douglas multiple-output production function (rSDF-CD). Specifically, DEA and rDEA performed well when data were generated using a linear functional form, whereas rSDF-CD performed well on Cobb-Douglas and piecewise Cobb-Douglas forms. In applied work an analyst would ideally select the efficiency measurement technique given the true underlying functional form of their data. However, identification of the true underlying functional form is difficult, if not impossible [1], and we expect this issue to be further compounded in the presence of inefficiency. Nevertheless, we used our simulation environment, where the functional form was known, to evaluate the performance of the following functional form tests: comparison of monotonicity violations, the Ramsey RESET test [2], and minimization of root mean square error (RMSE) in level and logarithmic space.

Following scenarios *d* and *g,* we generated data following linear (simulation 1) and Cobb-Douglas (simulation 2) multiple-output production functions, with multiplicative error, $e^{v_{i}}$ with $v_{i}\sim N\left( 0,{0.1}^{2} \right),$ applied to both inputs and outputs, and $unif(0,1)$ distributed efficiency. We ran each simulation scenario 2,000 times. For each iteration, we ran two regressions, one regression assuming a linear functional form (a) and another regression assuming a Cobb-Douglas functional form (b). These traditional least squares-based regression techniques failed to distinguish between noise and inefficiency, which could potentially lead to faulty conclusions [3]. With our simulation, however, we know rDEA is capable of accurately predicting efficiencies given a linear functional form and rSDF-CD is capable of accurately predicting efficiencies given a Cobb-Douglas functional form. As a result, if we adjust for inefficiencies, we can adequately compare regression (a) to (b). To do so, in each iteration, we removed inefficiencies by running regression (a) on data with outputs divided by rDEA-estimated efficiencies and similarly ran regression (b) on data with outputs divided by rSDF-CD-estimated efficiencies. Regressions (a) and (b) are shown below where $x_{ri}$ denotes the $r^{th}$ input, $y_{ji}^{*}$ denotes the $j^{th}$ output adjusted to its technically efficient level as estimated by rDEA and rSDF-CD, and $\varepsilon_{i}$ denotes ordinary least square error:

$y_{1i}^{*}= \beta_{0}+\sum_{\boldsymbol{r=1}}^{\boldsymbol{3}} x_{ri}\beta_{r}+\sum_{j=2}^{3} y_{ji}^{*}\alpha_{j}+ \varepsilon_{i}$ (a)

${ln(y}_{1i}^{*})= \beta_{0}+\sum_{r=1}^{3} ln(x_{ri})\beta_{r}+\sum_{j=2}^{3} ln\left( y_{ji}^{*}/y_{1i}^{*} \right)\alpha_{j}+ \varepsilon_{i}$ (b)

**Monotonicity violations**

Assuming decision making units (DMUs) are not experiencing decreasing returns to scale, we would expect an increase in inputs to result in an increase in $y_{1}^{*}$, with all else equal. Similarly, we would expect an increase in $y_{2}^{*}$ or $y_{3}^{*}$ to lead to a decrease in $y_{1}^{*}$, with all else equal. These assumptions can be validated in regression analyses by checking that the partial derivative of the regression with respect to any input (output) is positive (negative). By comparing the total number of monotonicity violations in regressions (a) and (b), we may be able to determine which functional form provides the best fit.

As shown in Table D, regressions (a) and (b) incurred the same number of monotonicity violations for 88% of iterations in simulation 1. Similarly, in simulation 2 regressions (a) and (b) resulted in the same number of violations in 98% of iterations. In this scenario, comparison of monotonicity violations did not differentiate between functional forms.

**Table D. Monotonicity violations.**

| **Indicator** | **Simulation 1** | **Simulation 2** |
| --- | --- | --- |
| Percent of iterations with regression (a) incurring fewer monotonicity violations than regression (b) | 0% | 0% |
| Percent of iterations with regression (b) incurring fewer monotonicity violations than regression (a) | 12% | 2% |
| Percent of iterations where regression (a) and (b) incurred the equivalent number of monotonicity violations | 88% | 98% |

**Ramsey RESET test**

The Ramsey RESET test examines whether non-linear combinations of fitted values explains the response variable significantly at the 5% level. For instance, if the test is successful in simulation 2, we expect that the Ramsey RESET quadratic term in regression (a) to be significant and the Ramsey RESET quadratic term in regression (b) to lack statistical significance. Based on this result, we would then reject the linear functional form postulated by regression (a). We would expect the opposite to hold true for simulation 1.

As seen in Table E, the Ramsey RESET test performed well in simulation 2, correctly rejecting the linear functional form in 80% of iterations (80% = 84% – 4%). However, in simulation 1, the Cobb-Douglas functional form was rejected in 9% of iterations (9% = 13% – 4%), and in 35% (35% = 39% –4%) of iterations regression (a) ‒ the correctly specified functional form ‒ was rejected. The Ramsey RESET test failed to discriminate between functional forms when the underlying functional form is linear.

**Table E. Ramsey RESET test.**

| **Indicator** | **Simulation 1** | **Simulation 2** |
| --- | --- | --- |
| Percent of iterations with Ramsey RESET test quadratic term significant at the 5% level in regression (a) | 39% | 84% |
| Percent of iterations with Ramsey RESET test quadratic term significant at the 5% level in regression (b) | 13% | 4% |
| Percent of iterations with Ramsey RESET test quadratic term significant at the 5% level in both regression (a) and (b) | 4% | 4% |

**Minimization of RMSE**

RMSE is a measure of in-sample fit. For each iteration we tested whether regression (a) or (b) produced a lower RMSE. We compared the RMSE in both level and logarithmic space since regression (a) and regression (b) are measured in each space, respectively.

Regression (a) predicted at least one value less than or equal to zero, which prevented a comparison of the RSME in logarithmic space in 93% of iterations in simulation 1 and 80% of iterations in simulation 2 (Table F). In level space, the RMSE for regression (b) was lower than the RMSE for regression (a) in 83% of iterations in simulation 1 and 100% of iterations in simulation 2 (Table G). Comparison of the RMSE in level space frequently led to incorrectly rejecting the linear functional form.

**Table F. Comparison of RMSE in logarithmic space.**

| **Indicator** | **Simulation 1** | **Simulation 2** |
| --- | --- | --- |
| Percent of iterations with RMSE lower in regression (a) than (b) | 0% | 0% |
| Percent of iterations with RMSE lower in regression (b) than (a) | 7% | 20% |
| Percent of iterations with at least one predicted value of regression (a) less than or equal to zero | 93% | 80% |

**Table G. Comparison of RMSE in level space.**

| **Indicator** | **Simulation 1** | **Simulation 2** |
| --- | --- | --- |
| Percent of iterations with RMSE lower in regression (a) than (b) | 17% | 0% |
| Percent of iterations with RMSE lower in regression (b) than (a) | 83% | 100% |

**References**

1. Griffin RC, Montgomery JM, Rister ME. Selecting Functional Form in Production Function Analysis. Western Journal of Agricultural Economics.

2. Ramsey JB. Tests for Specification Errors in Classical Linear Least-Squares Regression Analysis. Journal of the Royal Statistical Society Series B (Methodological). 1969;31: 350–371.

3. Kumbhakar SC, Lovell CAK. Stochastic Frontier Analysis. New York, NY, USA: Cambridge University Press; 2000.
