# Supplementary material for: Efficiency of Health Care Production in Low-Resource Settings: A Monte-Carlo Simulation to Compare the Performance of Data Envelopment Analysis, Stochastic Distance Functions, and an Ensemble Model
Source: PLoS One. 2016 Jan 26;11(1):e0147261. doi: 10.1371/journal.pone.0147261 (PMC4727806; doi:10.1371/journal.pone.0147261)
Supplement: S3 Appendix — Results on functional form (Table H) and inefficiency distributions (Table I) are included in this supplementary file. (DOCX) [file pone.0147261.s003.docx]

**S3 Appendix. Results for the single-output production function**

**Table H. Variations in the functional form for the single** **output case.**

| **Functional form** | **Model specification** | **Method** | **MAD** | **NOTFront** | $\mathbf{PU}_{\boldsymbol{20\%}}$ | $\mathbf{PO}_{\boldsymbol{20\%}}$ | $\boldsymbol{r}_{\boldsymbol{s}}$ |
| --- | --- | --- | --- | --- | --- | --- | --- |
| **Cobb-Douglas** | $\theta_{i}\sim unif\left( 0,1 \right)$ | DEA | 0.051 | 2.7% | **0.0%** | 22.4% | 0.967 |
|  |  | rDEA | 0.031 | 0.4% | 17.5% | 0.6% | 0.955 |
|  |  | rSDF-CD | **0.008** | **0.0%** | **0.0%** | **0.0%** | **0.998** |
|  |  | ENS | 0.017 | **0.0%** | 4.0% | 0.3% | 0.987 |

Note: Numbers in **bold** highlight the best outcome for each performance indicator across the alternative approaches. MAD: median absolute deviation, NOTFront: percentage of misclassified DMUs, ${PU}_{20\%}$: percentage of underestimation, ${PO}_{20\%}$: percentage of overestimation,$r_{s}$: Spearman’s rank correlation.

**Table I. Functional form, inefficiency distribution, and measurement error for single output cases.**

| **Inefficiency distribution** | **Model specification** | **Method** | **MAD** | **NOTFront** | $\mathbf{PU}_{\boldsymbol{20\%}}$ | $\mathbf{PO}_{\boldsymbol{20\%}}$ | $\boldsymbol{r}_{\boldsymbol{s}}$ |
| --- | --- | --- | --- | --- | --- | --- | --- |
| **Half-normally distributed inefficiency** | $u_{i}\sim N^{+}(0,{0.05}^{2})$, $v_{i}\sim N\left( 0,{0.02}^{2} \right)$ | DEA | 0.016 | **0.0%** | **0.0%** | **0.0%** | 0.617 |
|  |  | rDEA | 0.084 | **0.0%** | 24.8% | **0.0%** | 0.235 |
|  |  | rSDF-CD | **0.011** | **0.0%** | **0.0%** | **0.0%** | **0.791** |
|  |  | ENS | 0.044 | **0.0%** | 5.9% | **0.0%** | 0.350 |
|  | $u_{i}\sim N^{+}(0,{0.2}^{2})$, $v_{i}\sim N\left( 0,{0.08}^{2} \right)$ | DEA | 0.052 | 1.0% | 1.1% | 3.4% | 0.675 |
|  |  | rDEA | 0.123 | 0.1% | 35.0% | **0.2%** | 0.537 |
|  |  | rSDF-CD | **0.038** | **0.0%** | **0.2%** | 1.1% | **0.791** |
|  |  | ENS | 0.069 | **0.0%** | 10.4% | **0.2%** | 0.663 |
| **Uniformly distributed inefficiency** | $u_{i}\sim unif\left( 0,1 \right)$, $v_{i}\sim N\left( 0,{0.05}^{2} \right)$ | DEA | 0.044 | 2.8% | **0.0%** | 24.1% | 0.961 |
|  |  | rDEA | 0.036 | 0.4% | 20.2% | 1.3% | 0.950 |
|  |  | rSDF-CD | **0.020** | **0.0%** | 1.3% | 0.5% | **0.990** |
|  |  | ENS | 0.025 | **0.0%** | 7.7% | **0.4%** | 0.981 |
|  | $u_{i}\sim unif\left( 0,1 \right)$,$v_{i}\sim N\left( 0,{0.20}^{2} \right)$ | DEA | **0.065** | 3.4% | **17.6%** | 23.0% | 0.903 |
|  |  | rDEA | 0.090 | 0.7% | 51.7% | 5.0% | 0.900 |
|  |  | rSDF-CD | 0.071 | **0.0%** | 39.0% | 5.2% | **0.935** |
|  |  | ENS | 0.077 | **0.0%** | 46.2% | **3.9%** | 0.930 |

Note: Numbers in **bold** highlight the best outcome for each performance indicator across the alternative approaches. MAD: median absolute deviation, NOTFront: percentage of misclassified DMUs, ${PU}_{20\%}$: percentage of underestimation, ${PO}_{20\%}$: percentage of overestimation,$r_{s}$: Spearman’s rank correlation.
